# Cytologic features of metanephric adenoma of the kidney

**DOI:** 10.4103/1742-6413.49164

**Published:** 2009-04-04

**Authors:** José A. Jiménez-Heffernan, Eva Tejerina, Pilar González-Peramato, Blanca Vicandi, Ana López-García

**Affiliations:** 1 Departments of Pathology, University Hospitals, Puerta de Hierro, Madrid, Spain; 2 Departments of Pathology, University Hospitals, La Paz, Madrid, Spain

To the Editor,

Metanephric adenoma (MA) is a rare, benign renal neoplasm that consists of a proliferation of small epithelial cells, which form acinar, tubular, papillary and glomeruloid structures. The first large series describing the tumor were reported in 1995.[1,2] In many cases, the benign behavior, well-defined limits and small size of the tumors, make them candidates of conservative surgical management. That only a few cytological descriptions are available[3–9] is a reflection on the rarity of the tumor [Table 1]. We describe a case evaluated intraoperatively and in which cytology allowed a precise recognition.

**Table 1:** Reported cytologic cases of metanephric adenoma

	*Number of cases*	*Cytologic material*	*Cytologic diagnosis*
Zafar *et al*.[3]	2	FNA	Suggestive of WT and embryonal adenoma
Renshaw *et al*.[4]	1	Intraoperative	-
Granter *et al*.[5]	2	FNA	-
Xu *et al*.[6]	1	FNA	Low grade neoplasm (MA vs WT)
Khayyata *et al*.[7]	1	FNA	MA
Bosco *et al*.[8]	1	FNA	MA
Portugal *et al*.[9]	1	FNA	MA vs WT
Present case	1	Intraoperative	Suggestive of MA

A 56-year-old woman, presented with a 3 cm renal tumor. Tumorectomy with intraoperative evaluation was performed. The renal mass was well defined, solid and had a grey to white color. Air-dried and alcohol-fixed cytologic samples were obtained after scraping the tumor surface. The smears showed a monomorphous cellular population in a clean background. Most cells presented as naked nuclei, with oval to round morphology. They distributed as clusters and single cells. A minimum amount of deeply stained cytoplasm was evident only in a few aggregates. Many clusters showed an evident tubular arrangement [Figure 1a]. Small, three-dimensional, tightly packed morules were present with a smooth outer surface [Figure 1b]. Ribbons, glandular [Figure 2a] and pseudo-papillary structures were also present. The nuclei were slightly oval and monomorphic [Figure 2b]. Chromatin was regularly distributed and the nuclear membrane was smooth; no nucleolus was seen. The smears showed no atypia, mitotic figures, necrosis or crushing chromatin artefact. Histology revealed an un-encapsulated neoplasm, with a predominant acinar and tubular growth, with pseudo-papillary and solid areas and inconspicuous stroma [Figures. 3a and b]. Glomeruloid structures were also seen. The cells were small, oval, and uniform with scanty cytoplasm. No nuclear atypia, mitotic figures or apoptosis were seen. Tumoral stroma was inconspicuous, with no necrosis. Immunohistochemistry revealed expression of vimentin, CD57 and WT1. There was no expression of cytokeratin 7 or epithelial membrane antigen.

**Figure 1:**
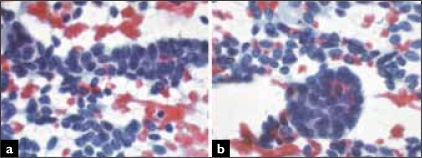
Single cells and clusters with tubular configuration (A) and morules (B) (Papanicolaou).

**Figure 2:**
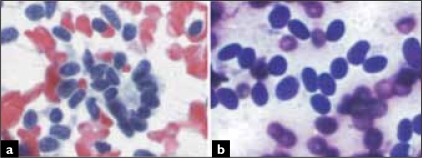
Glandular structures were also evident. Chromatin is regularly distributed and there are no relevant nucleoli (A, Papanicolaou). Cells have inconspicuous cytoplasm and predominantly oval, monomorphic nuclei (B, Diff Quik).

**Figure 3:**
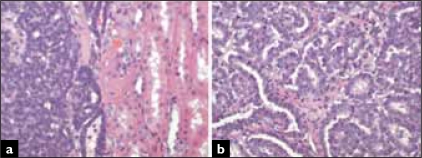
Histologically, the limit with normal parenchyma is clear cut, with no capsule (A). Cells are small, with a variable growth pattern in which tubular and pseudo papillary structures predominate (B) (Hematoxylin and eosin).

In our opinion, the histopathologic features of MA are well reflected on cytology, allowing its recognition. The present case, as well as previously published ones, reveal a similar cytologic pattern that can be summarized as a small blue cell, epithelial tumor, with oval cells and no atypia. It shows different epithelial structures with tubules, glands, pseudopapillae and morules. In our case, the presence of numerous single cells is probably related to the scraping of the tumor, since fine needle aspirate samples show a marked predominance of clusters.[2,3,5–9] This cytologic image differs considerably from that of other adult renal tumors. The extensive use of image studies has resulted in the detection of many small renal masses.

Therapeutic decisions partially depend on the histologic nature of the tumor, but obtaining this information preoperatively may be difficult. Fine needle aspiration and intraoperative studies are often requested for aiding in decision making.

The present case illustrates the utility of cytologic evaluation of intraoperative tissue samples. Frozen sections raised the possibility of papillary renal cell carcinoma. This is an obvious consideration in the differential diagnosis of MA, since both neoplasms may share histologic features.[1,2] In our case, papillary renal cell carcinoma was not considered after cytologic evaluation. Instead, the small cell size and epithelial nature raised the possibilities of nephroblastoma and MA. Cellular monomorphism, absence of stromal component, extensive epithelial differentiation, oval morphology, absence of necrosis and apoptosis, as well as the patient's age, favored the possibility of MA. Epithelial-predominant nephroblastoma may resemble MA more closely. In fact, there are also immunohistochemical similarities, and, for some authors, MA represents a hyperdifferentiated, mature form of nephroblastoma.[10]

Papillary carcinoma and MA may share architectural features. Tubular, pseudo-papillary structures and spherules of tumoral cells can be present in both neoplasms.[1] However, the neoplastic cells differ. Those from papillary carcinoma are larger and show moderate amounts of cytoplasms, sometimes vacuolated or containing hemosiderin. The macrophagic cell population so frequently present in papillary carcinoma is rare in MA.

In conclusion, cytology, either as fine needle aspirates or during intraoperative procedures, may be of great value in the recognition of MA.

## COMPETING INTEREST STATEMENT BY ALL AUTHORS

No competing interest to declare by any of the authors.

## AUTHORS' CONTRIBUTIONS

JAJH, Conceptual organization, participated in the intraoperative diagnosis and writing of manuscript

ET, Participated in the intraoperative and histopathologic diagnosis and manuscript review

PGP and BV, Cytologic review of the case, differential diagnosis and manuscript review

AL, Conceptual organization, cyto-histologic evaluation and manuscript review
